# The Effects of an Online Behavioral Activation Program with Informational Support Messages on Patients with Complex Regional Pain Syndrome

**DOI:** 10.1192/j.eurpsy.2025.1892

**Published:** 2025-08-26

**Authors:** M. Jung, S. Cho

**Affiliations:** 1 Department of Psychology, Chungnam National University, Daejeon, Korea, Republic Of

## Abstract

**Introduction:**

Complex Regional Pain Syndrome (CRPS) is a chronic neuropathic pain condition that significantly reduces patients’ quality of life. Few studies have examined the application of short-term Behavioral Activation (BA) programs for patients with CRPS, and studies that include informational support messages are scarce.

**Objectives:**

This study is intended to investigate the effects of an online BA program, accompanied by informational support messages, on pain intensity, pain interference, pain catastrophizing, depression, life satisfaction, and behavioral patterns in patients with CRPS.

**Methods:**

Two patients with CRPS participated in an eight-session online BA program using a multiple-baseline design. After the first session, participants completed daily activity monitoring sheets, and the baseline was measured. The BA intervention began in the third session, and from that point until the eighth session, participants received immediate informational support messages once they completed their monitoring sheets. The informational support messages consisted of graphs comparing the previous day’s pain intensity, depression levels, and activity levels, as well as linear trendline graphs based on recorded data. Additionally, questionnaires were used to measure pain intensity, pain interference, pain catastrophizing, depression, life satisfaction, and behavioral patterns pre-intervention, post-intervention, and at a four-week follow-up, and effect sizes (Cohen’s d) were calculated.

**Results:**

Daily activity monitoring sheets indicated that activity levels significantly increased during the intervention phase and were maintained or further increased at follow-up. Depression levels gradually decreased from the intervention phase, but pain intensity showed no significant change.

Questionnaires revealed that pain catastrophizing and depression decreased post-intervention and at follow-up compared to baseline, while life satisfaction increased, and pain avoidance behaviors decreased. However, pain intensity increased compared to baseline, and pain interference decreased at follow-up. Effect sizes, measured by Cohen’s d, indicated large effects for all variables except behavioral patterns and pain interference post-intervention, and for all variables except behavioral patterns at follow-up.

**Conclusions:**

These findings suggest that an online BA program with informational support may be effective for patients with CRPS.

**Disclosure of Interest:**

M. Jung Grant / Research support from: National Research Foundation of Korea Grant funded by the Korean Government (NRF-2022S1A5A2A03050752), S. Cho Grant / Research support from: National Research Foundation of Korea Grant funded by the Korean Government (NRF-2022S1A5A2A03050752)

